# Knowledge, Awareness, and Attitude in Using Dental Implants as an Option in Replacing Missing Teeth Among Dental Patients: Survey-Based Research in a Dental Teaching Hospital in Derabassi, Punjab

**DOI:** 10.7759/cureus.27127

**Published:** 2022-07-21

**Authors:** Khyati Arora, Navneet Kaur, Gurpreet Kaur, Umesh Garg

**Affiliations:** 1 Department of Periodontics, National Dental College & Hospital, Derabassi, IND; 2 Department of Periodontics & Implantology, National Dental College & Hospital, Derabassi, IND

**Keywords:** patient education, implant-supported prosthesis, edentulous patients, missing teeth, dental patients, patient satisfaction, patient opinion, dental implants

## Abstract

Background

Among many options that are available to replace missing teeth, dental implants are very popular. Information about patient awareness of dental implants is an important parameter for planning health care services and marketing. Knowledge of the treatment method minimizes any negative image that can be caused by a lack of proper communication.

Aim and objective

The objective of this study is to assess and obtain information about the knowledge, awareness, and attitude in using dental implants as an option for replacing missing teeth among dental patients.

Materials and methods

A questionnaire survey was conducted on 5000 subjects. This was a self-administered two-part questionnaire consisting of 18 questions. The percentage response for each question from all participants was obtained and the data collected was calculated and analyzed using Statistical Package for Social Sciences (SPSS) software version 21.0 (IBM Inc., Armonk, New York).

Results

Out of 5000 patients, 75% were already aware of dental implants from various means (dentist, family friends, social media such as television, radio, newspaper, or magazine). Based on the survey results, a significant number of patients had knowledge and awareness as well as a positive attitude towards using dental implants as an option for missing teeth.

Conclusion

People have a decent to fair level of understanding and awareness of dental implants, which are used to replace lost teeth. The dentist plays the most important role in this regard, and this can be accomplished by implementing patient education programs and counseling centers on the usage and benefits of dental implants.

## Introduction

Tooth loss is a frequent condition that can occur due to dental caries, periodontal disease, facial trauma, endodontic failure, and occasionally iatrogenic factors [1]. Tooth loss can be traumatic and upsetting, and it is considered a serious life event that requires remarkable social and psychological readjustment [2]. The World Health Organization classifies edentulous people as physically impaired due to the loss of an important part of their body [1].

The complications usually seen in edentulous patients are chewing and masticatory deficiency, phonetic problems, loss of facial support and esthetics [3] as well as social embarrassment [4]. Traditionally, missing teeth are replaced by removable partial dentures, fixed partial dentures (bridges), and complete dentures in cases of complete edentulousness [2, 5]. Implants offer alternative treatment options to fully or partially edentulous patients. Lack of sufficient communication may result in a negative image, which may be avoided if individuals are informed about tooth replacement [6]. Factors affecting the choice of one of these treatments include aesthetics, number of missing teeth, either anterior or posterior teeth, financial status, quality of ridge and alveolar bone, age, gender, socioeconomic conditions, and patient's choice [3].

Dental implants were originally used to treat edentulous patients and have been linked to better denture retention, stability, and functional efficiency [7,8]. The dental implant is into the alveolar bone, where it resembles a tooth root and acts as an anchor for an abutment that provides support, retention, and stability to the superstructures of dental prostheses. A predictable higher rate of osseointegration was first reported in the 1980s, and thereafter studies shifted towards investigating the aesthetic requirements of implant restoration and extending their clinical applications from single tooth replacement to partial and complete replacement of teeth; management of orofacial defects; rehabilitation of compromised oncological patients; and as anchor points in orthodontics [9]. The willingness to undergo implant treatment and its success depends on the knowledge and expectations of the patients as well as the care, skill, and judgment of the practitioner [6]. It has become increasingly popular as the majority of the patients treated with implant-supported prostheses have reported improvement in their quality of life, assurance, self-confidence, including psychological benefits, and conservation of tooth structure adjacent to the teeth to be replaced [10].

Implant-supported prostheses have shown benefits such as greater masticatory efficiency, alveolar bone maintenance, better function, phonetics, and aesthetics [6]. Due to its high success rates and predictability, its clinical implication is increasing rapidly [10]. Information about dental implants can be provided through various channels and techniques.

An implant retentive prosthesis can be the best treatment choice for partially or completely edentulous patients, but there is limited availability of implant therapy in our part of the world [11]. However, treatment decisions should not be made based on clinical examination or dentist's consultation only, but rather it should be according to the patient's choice and priority. In most cases, the final decision-making depends on financial status, level of education, awareness, and knowledge about various treatment modalities available for the patients.

Hence, the present study was conducted to evaluate the knowledge, attitude, and awareness of patients toward dental implants as an option for replacing missing teeth.

## Materials and methods

The present cross-sectional questionnaire-based survey was conducted among 5000 dental patients from June 2021 to January 2022 in a six-month survey. An information sheet regarding the survey was provided to all the dental patients in the preferred language (English, Hindi, Punjabi), and verbal consent was obtained from all the participants before the start of the study.

The main objective of the survey was to assess the awareness, attitude, need, and demand for the replacement of missing teeth made by the participants. We also attempted to assess the socio-demographic variables such as age, gender, and the educational level of the participants.

Being a partially dented patient and above 18 years of age were considered as the inclusion criteria. Physically handicapped patients and those from dental-related professions/occupations (dental surgeons, dental assistants, dental hygienists, dental students, and dental technicians) and fully edentulous patients were excluded from the survey sample. The dental patients were included from the outpatient department of periodontics and oral implantology of the National Dental College & Hospital, Derabassi. Ethical approval for the study was obtained from the institutional Ethical Board Committee at National College and Hospital, Derabassi, Punjab.

Questionnaire design

The data of the survey was collected through a structured interview in the form of a questionnaire based on previous research conducted by Tepper et al. and Rustemeyer et al [12, 13].

The questionnaire performance was modified according to the study population. The questionnaire is composed of 18 closed-ended questions based on two sections, namely (a) socio-demographic data, and (b) knowledge, awareness, and attitude regarding dental implants that include questions regarding the history of tooth extraction, sources of information about implants, choice of replacement of missing teeth and acceptance of dental implant as a treatment option.

The questionnaire was handed to the respective dental patients in the outpatient department of the department of periodontics and oral implantology during the routine visit to the dental patients.

Statistical analysis

The filled responses were then transferred to a Microsoft Excel sheet (Microsoft, Redmond, Washington) for appropriate statistical analysis. The percentage response for each question from all participants was obtained, and a test of percentage distribution was performed. The percentage was calculated, and the data was displayed using pie charts. The data was calculated and analyzed using Statistical Package for Social Sciences (SPSS) software version 21.0 (IBM Inc., Armonk, New York). 

## Results

Out of 5000 patients, 4800 patients responded positively by participating in this study. In this way, the response rate was 96%. The rest of the people didn't complete the questionnaire, and incomplete data were excluded from the survey.

Socio-demographic features of the study participants

**Figure 1:**
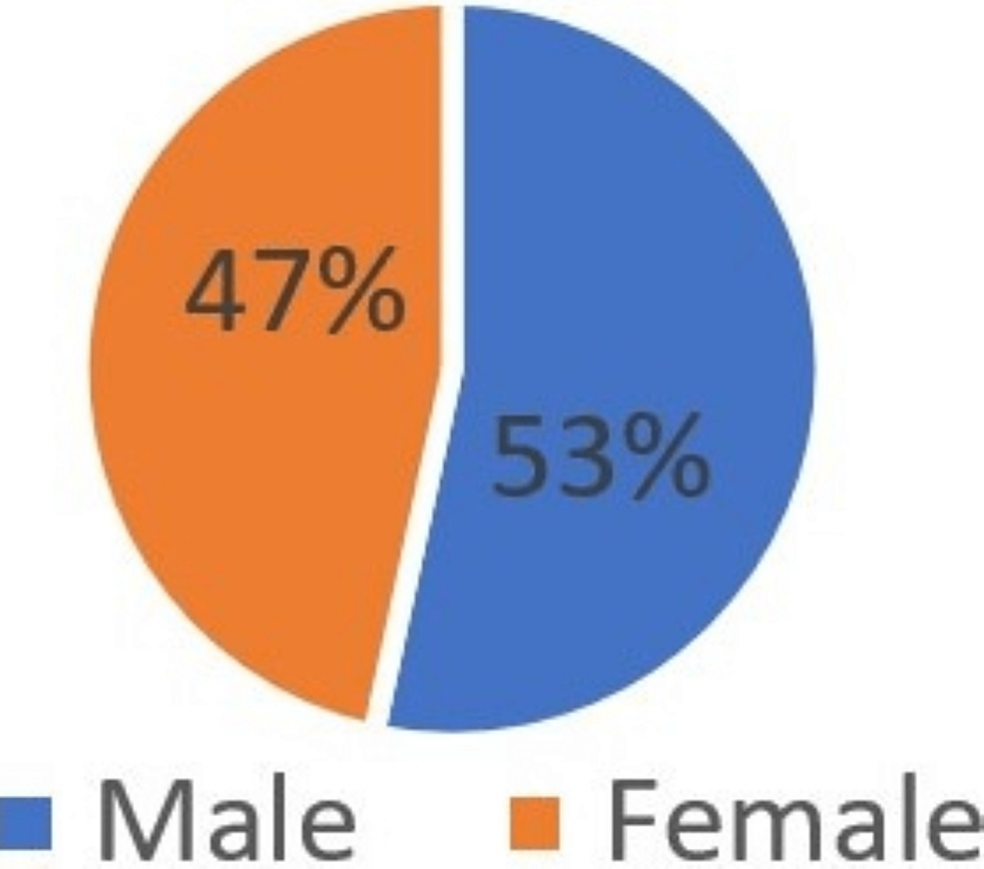
Gender distribution

**Figure 2:**
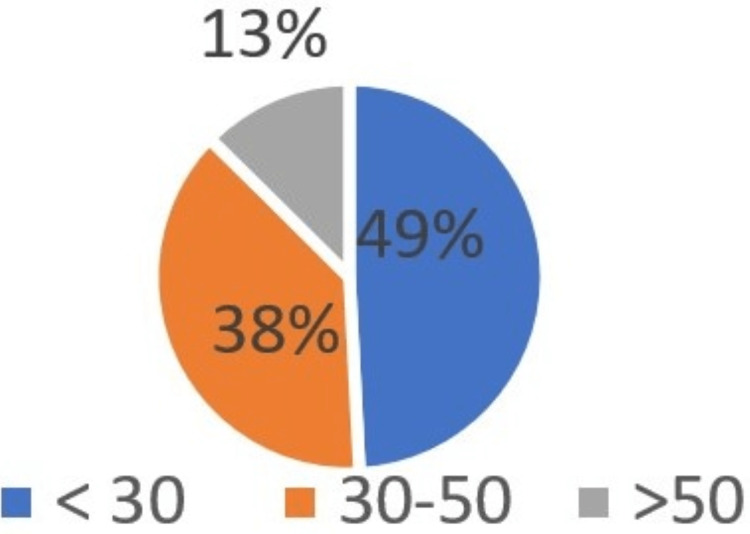
Age distribution

**Figure 3:**
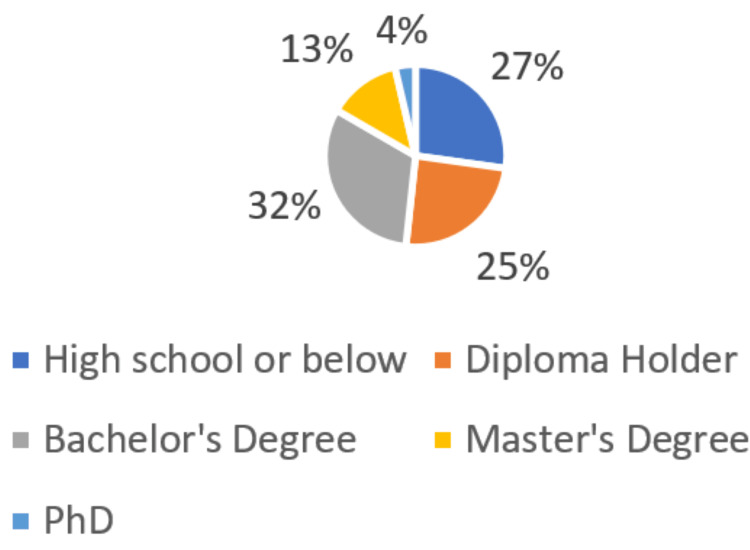
Education level

According to gender, 4800 samples included 2562 males (53.3%) and 2238 females (46.6%) (Figure 1). Male participants were slightly more predominant than females. The majority (49%) of the total sample were young participants below the age of 30 years. More than one-third of them (38.2%) of the total sample was in the age group of 30-50 years (Figure 2). Patient education status was also assessed. 31.6% of the patients have a bachelor's degree, and 27.2% were of secondary or lower educational level (Figure 3). A summary of the socio-dedmographic features of the study participants can be found in Table 1.

**Table 1:** Distribution of the study population according to socio-demographic variables (n=4800)

Variables		Subjects (n)	Percentage (%)
Age	<30 yrs	2360	49.2%
	30-50 yrs	1835	38.2%
	>50 yrs	604	12.6%
Gender	Male	2562	53.4%
	Female	2238	46.6%
Education level	High school or below	1306	27.2%
	Diploma holder	1176	24.5%
	Bachelor’s degree	1519	31.6%
	Master’s degree	624	13.0%
	PhD	175	3.6%

Survey questions on patients' knowledge and attitude regarding dental implants

When asked about the history of tooth extraction, more than half of the patients (66.7%) have a history of tooth extraction, while 3.6% did not remember anything regarding the history of tooth extraction. When the main reason for the tooth extraction was analyzed, 50.7% agreed that tooth decay or dental caries is the main reason which is followed by accident/trauma (24.4%) and gum diseases (21.6%). Regarding the time event of tooth extraction, 31.9% of patients positively agreed that the tooth was extracted between more than five years and less than 10 years (Table 2).

**Table 2:** Survey questions on patients' knowledge and attitude regarding dental implants (% of positive response) RPD: removable partial denture; FPD: fixed partial denture

Questions	Response	Subjects (N)	Percentage (%age)
Do you have any history of extraction?	Yes	3201	66.7
	No	1426	29.7
	Can’t say	173	3.6
If yes, what was the reason for extraction/loss of tooth?	Tooth decay	1651	50.7
	Gum disease	702	21.6
	Accident/trauma	793	24.4
	Other	110	3.4
When did you get the tooth extracted?	0-5 years	1038	31.9
	5-10 years	1021	31.3
	10-15 years	569	17.5
	>15 years	630	19.3
Do you want to replace the missing tooth?	Yes	2714	83.4
	No	540	16.6
What replacement do you need in the extracted tooth region?	RPD	425	13
	FPD	635	19.5
	Implants	1509	46.3
	Don’t know	691	21.2
Have you ever heard anything related to implants?	Yes	3585	74.8
	No	1205	25.2
What was your first source of information about implants?	Dentist	1650	34.5
	Relatives & friends	1187	24.9
	Family doctor/physician	737	15.4
	Television/radio	367	7.7
	Internet	589	12.3
	Newspaper/magazine/pamphlets	115	2.4
	Multimedia	131	2.7
What are the reasons for not taking dental implant as a treatment option for replacing missing teeth?	Very expensive treatment	2105	44
	Takes long time	1031	21.5
	Fear/anxiety	647	13.5
	Needs surgery	514	10.7
	Lack of information about implants	490	10.2

**Figure 4:**
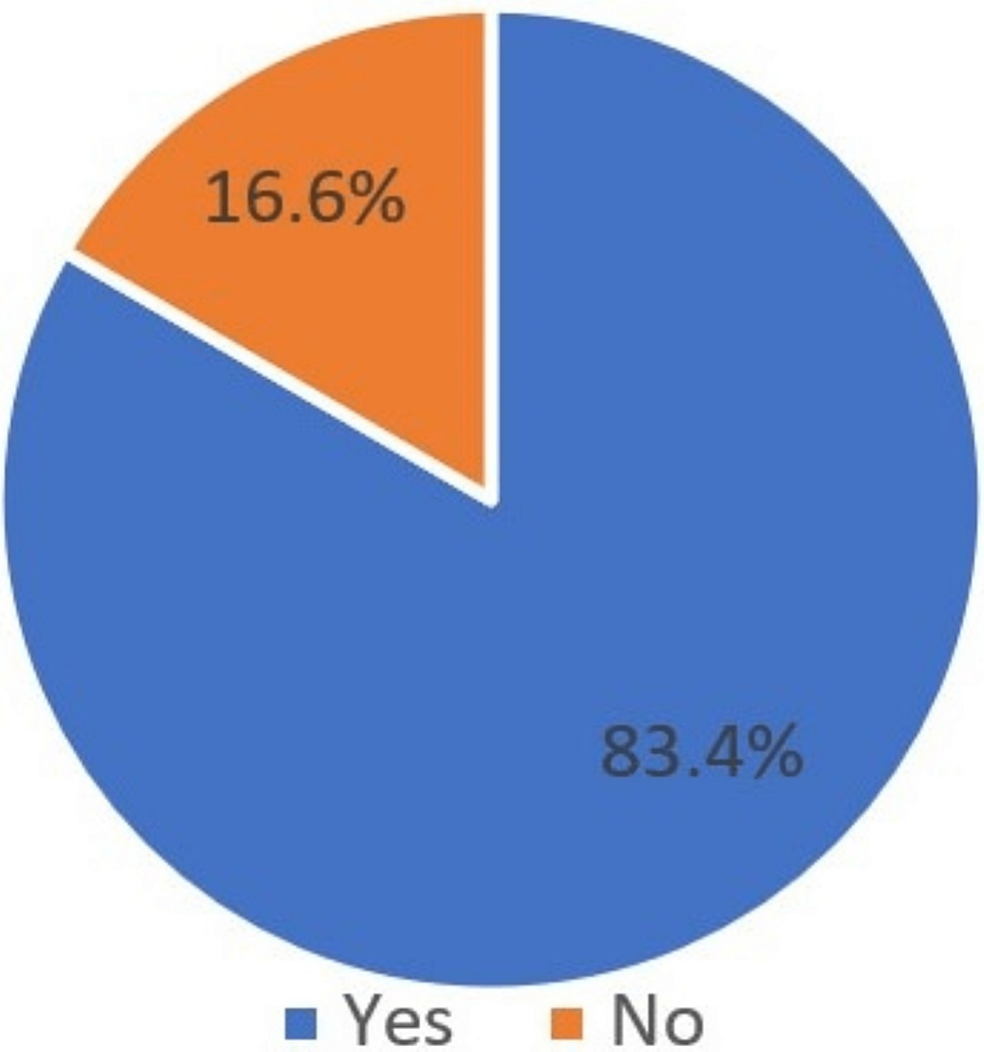
Replace missing tooth

**Figure 5:**
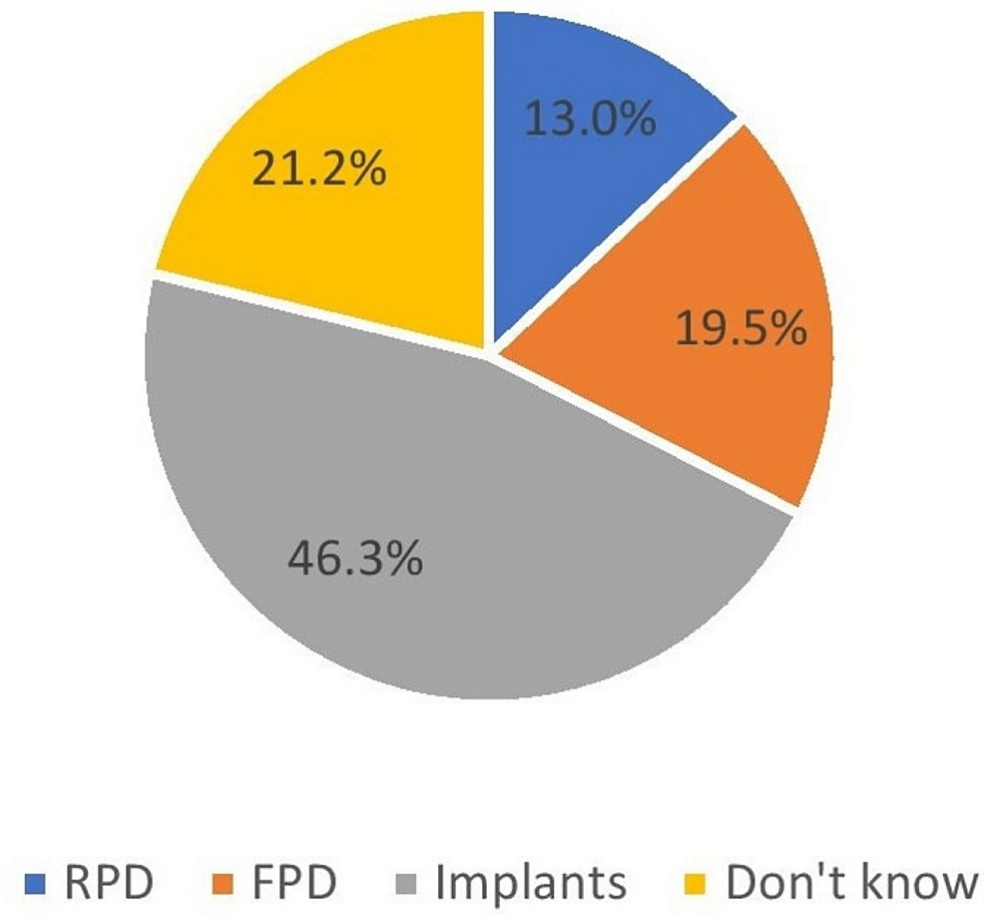
Replacement needed RPD: removable partial denture; FPD: fixed partial denture

The majority of the patients (83.4%) positively agreed that they want the replacement of the missing tooth (Figure 4). When asked about the type of replacement of missing teeth, more than half of the patients (46.3%) have an affirmative attitude toward dental implants as an option. However, there was an equivalent response for both removable partial denture (RPD) and fixed partial denture (FPD), which is 13% and 19.5%, respectively (Figure 5).

**Figure 6:**
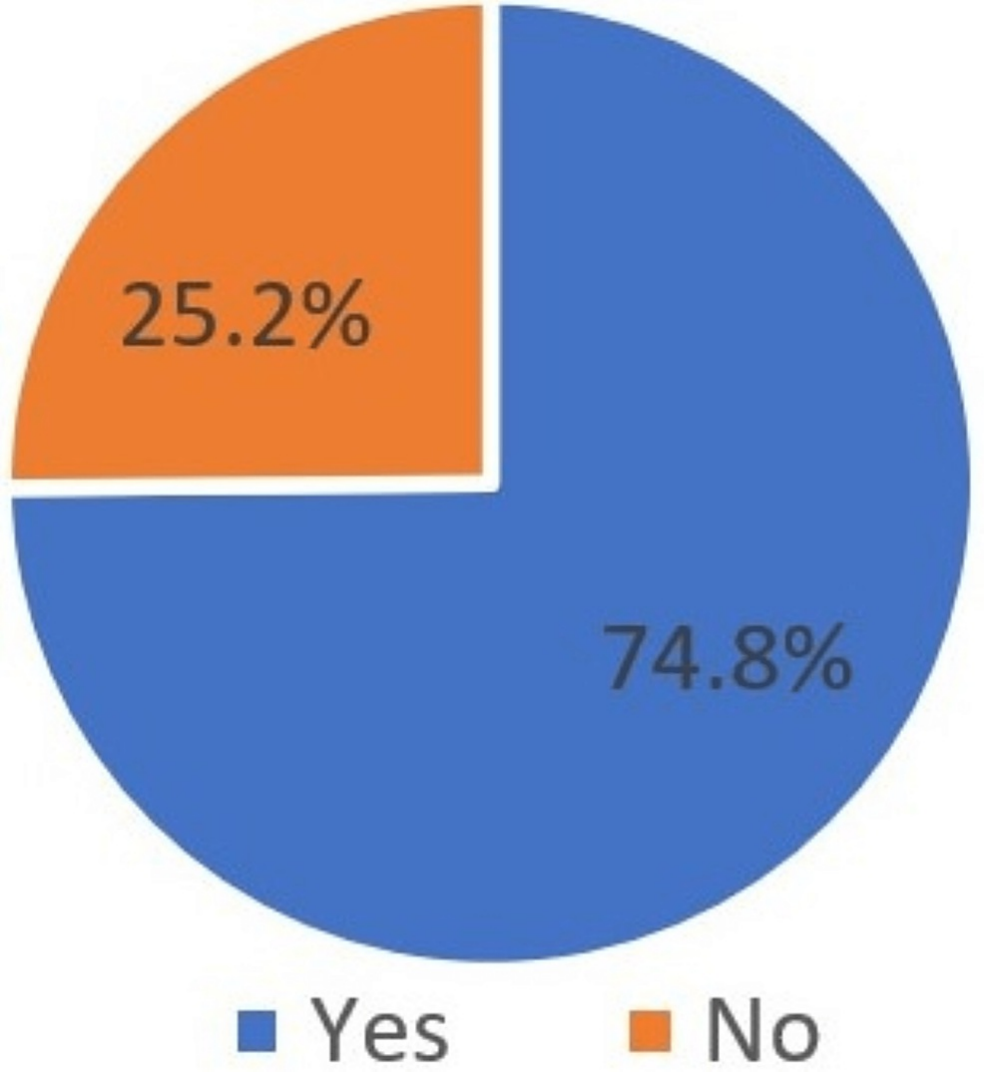
Heard about implants

Among the dental patients, more than half (74.8%) indicated having heard of dental implants, while 25.2% of respondents did not know anything about dental implants. According to this result, the rest of the results were from those participants who have heard about dental implants (Figure 6).

**Figure 7:**
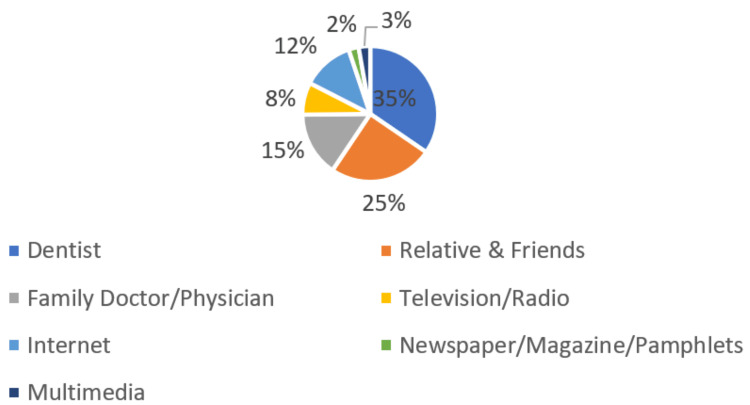
Source of information

When dental patients were investigated about their source of information for dental implants, it was dentists (34.5%), followed by relatives and friends (24.9%), then family doctor/physician (15.4%), and the least were television/radio (7.7%), newspaper and magazines (2.4%) and multimedia (2.7%) (Figure 7).

Regarding the unwillingness of the patient to replace the missing teeth with dental implants, the majority of the participants (44%) believed that dental implant treatment is very expensive treatment while 21.5% thought the reason is that dental implant treatment takes a longer time to complete and 13.5% thought that the reason is fear and anxiety, as well as implant treatment, may need surgery.

Survey questions on patients' awareness regarding dental implants

**Figure 8:**
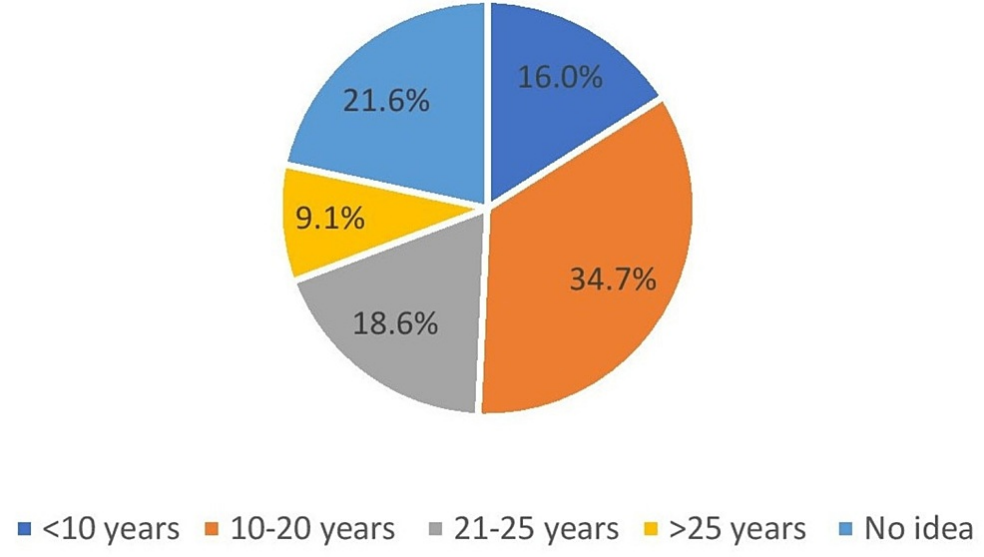
Functional life of implants

According to the results of the survey (Table 3), the majority of the patients were aware of implants and have a positive attitude towards them. 34.7% of patients believed that the functional life of implants is between 10-20 years, while 21.6% did not know the functional life of dental implants. 18.6% of the patients thought it will last up to 25 years, followed by 16% for less than 10 years of functional life of dental implants (Figure 8).

**Table 3:** Survey questions on patients' awareness regarding dental implants (% of positive response)

Questions	Response	Subjects (n)	Percentage (%)
According to you, what do you estimate as the functional life of implants?	<10 years	769	16.0
	10-20 years	1665	34.7
	21-25 years	891	18.6
	>25 years	438	9.1
	No idea	1037	21.6
Up to which amount are you prepared to pay as an additional payment for implant?	5000	1067	22.3
	10,000-20,000	1677	35.1
	>20,000	1014	21.2
	Varies from brand to brand (commercial availability of implant)	1019	21.3
What do you anticipate oral hygiene for the care of implants as compared with natural teeth?	More than natural teeth	1335	27.8
	Less than natural teeth	1331	27.7
	Similar to natural teeth	1243	25.9
	Can’t say	891	18.6
What are you expecting after getting treatment with implant as an option of missing teeth?	Long-lasting	2153	45.0
	Better esthetics	1830	38.2
	Less maintenance	804	16.8
Would you be interested in knowing more about dental implants?	Yes	3401	70.9
	No	1399	29.1
Where do you think implants are anchored?	Gums	809	16.9
	Teeth	1009	21.0
	Jawbone	1898	39.6
	Can’t say	1080	22.5
Would you go ahead and restore missing teeth with dental implants?	Yes	3298	68.8
	No	1499	31.2
Have you ever heard about experiences with implants from your friends?	Yes	2855	59.5
	No	1945	40.5
If yes, how successful was the implant?	Successful	1171	41.0
	Partially successful	1228	43.0
	Not successful	428	15.0
Have you ever heard about effects of dental implants on systemic health?	Yes	1792	37.3
	No	2008	41.8
	Can’t say	1000	20.8

The majority of the participants believed that dental implant treatment is one of the most expensive dental procedure and 35.1% of patients positively agreed that dental implant treatment cost up to 10000 to 20000. However, 21% of the patients believed that the cost of dental implant treatment may be more than 20000 or vary from brand to brand.

**Figure 9:**
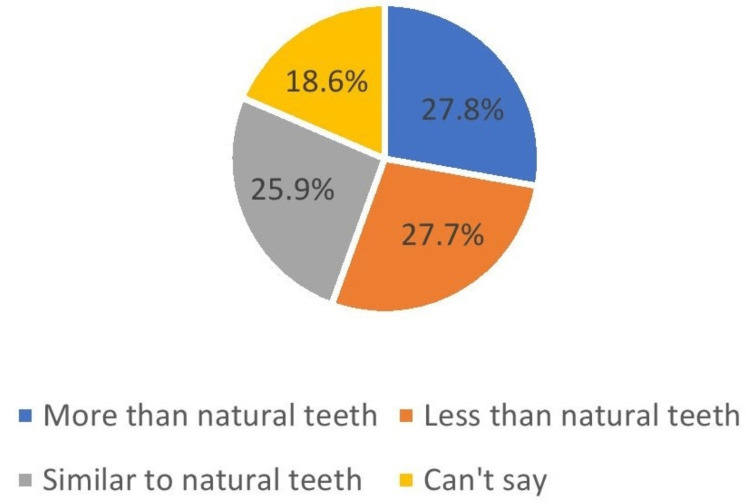
Anticipate oral hygiene

Regarding implant care and hygiene compared to natural teeth, 27.7% of participants thought that an implant might require more or less care and maintenance of oral hygiene than natural teeth. Moreover, 25.9% of participants believed that dental implants may require similar oral hygiene care as compared to natural teeth (Figure 9).

Regarding patients' expectations after getting a dental implant, the majority of surveyed participants (45%) believed that dental implant treatment as an option for missing teeth is long-lasting. Even 38.2% of surveyed participants positively believed that it is for better aesthetics and 16.8% expected dental implant treatment as an option for missing teeth for less maintenance as compared to natural teeth.

Among the participants, the majority (70.9%) indicated having more interest in knowing about dental implants, while 29.1% of participants did not show any interest in dental implants.

**Figure 10:**
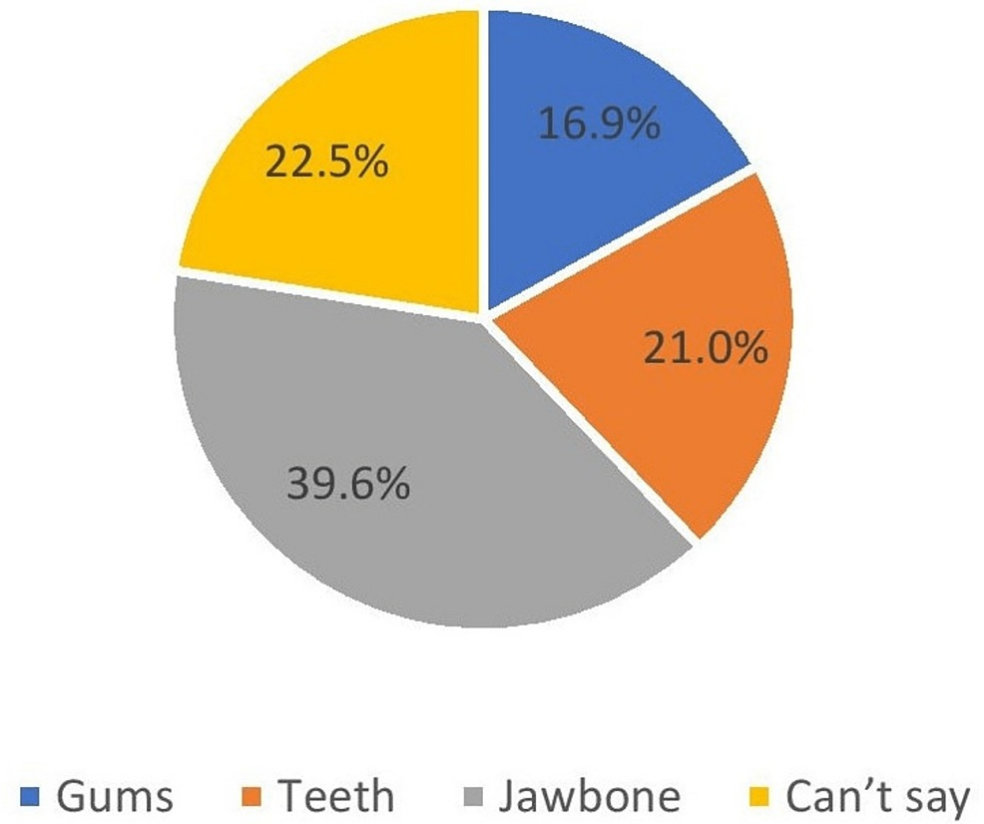
Where implants are anchored

More than one-third of the participants (39.6%) thought that dental implants are anchored in the jawbone, while 21% believed that they are anchored in the teeth, and 16.9% of participants thought that it is anchored in the gums. However, 22.5% of participants did not have any idea regarding the anchoring of dental implants into the oral cavity (Figure 10).

**Figure 11:**
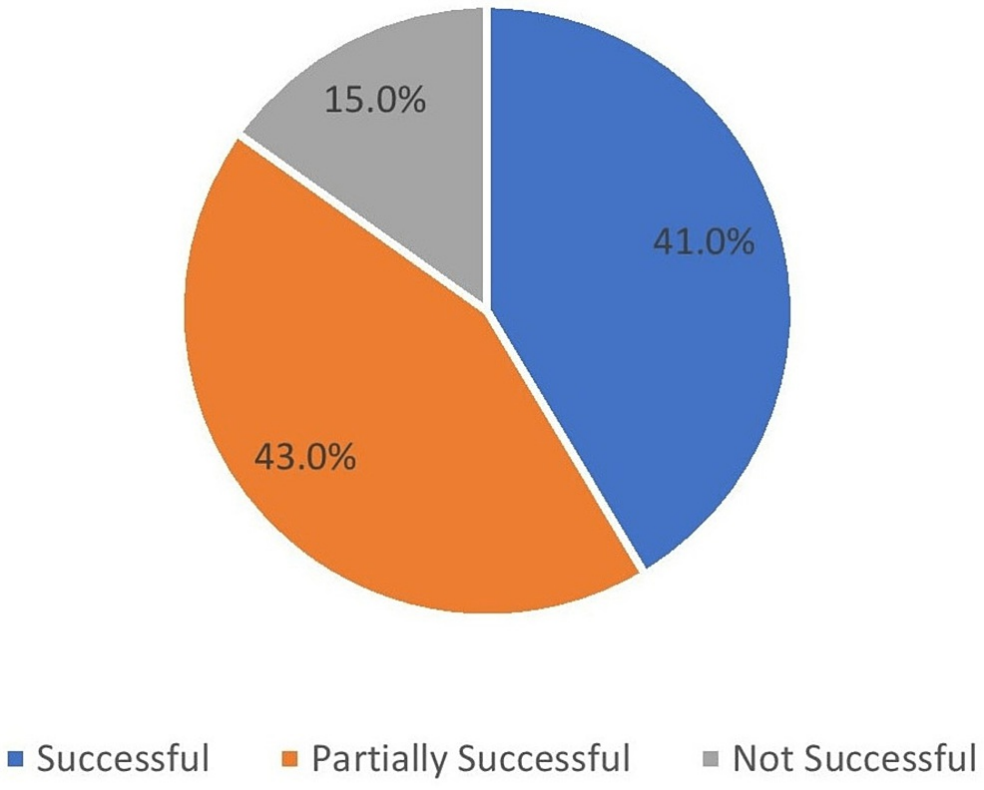
Success of implants

**Figure 12:**
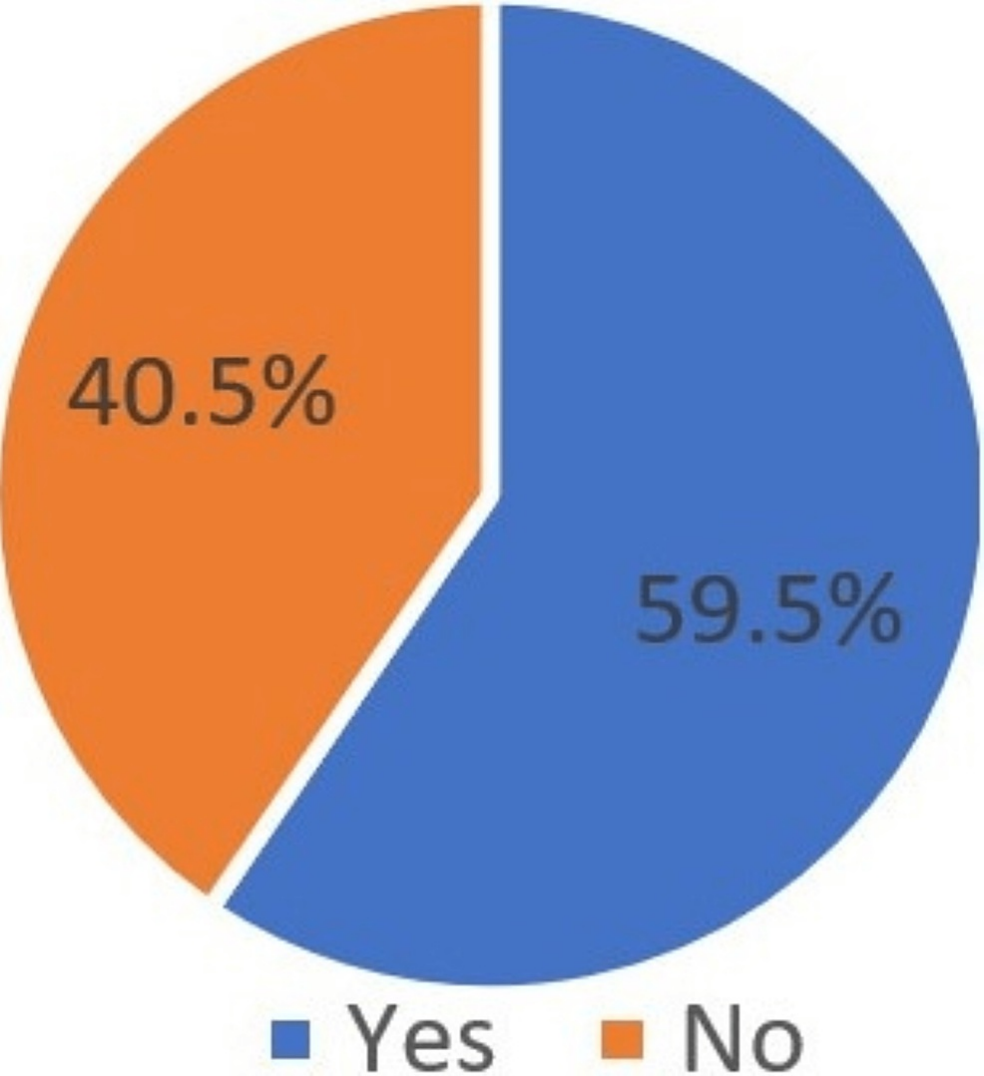
Heard of implants experience

The majority of the surveyed participants (68.8%) believed and filled the option as a yes that they will restore the missing teeth with dental implants in the future because they heard about the experience of dental implant treatment from their friends, and it was successful (Figure 11, 12).

**Figure 13:**
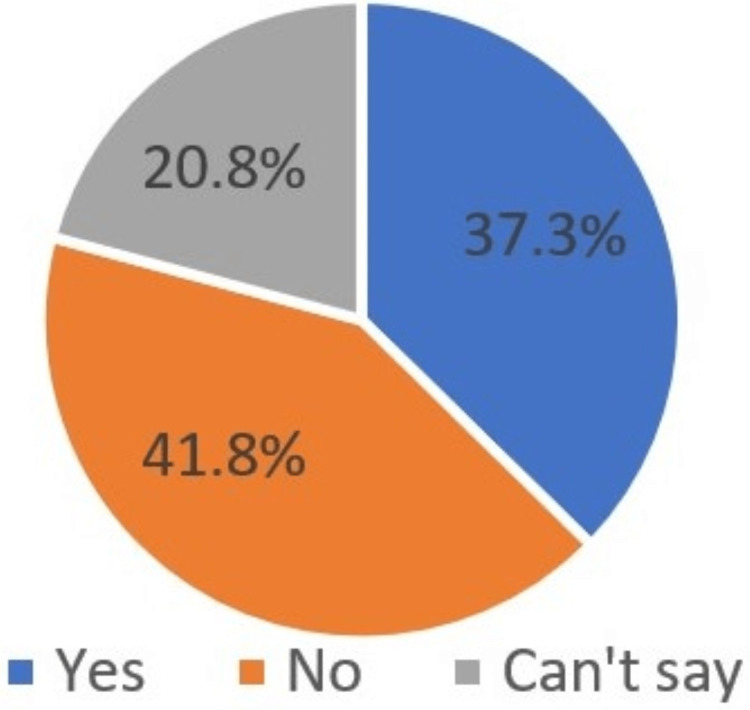
Heard about effects of dental implants

The majority of the participants (41.8%) believed that they have not heard of any effect of dental implants on systemic health, while 37.3% considered the effect of implant therapy on systemic health, and 20.8% were not aware of any such effects (Figure 13).

## Discussion

This study was conducted among dental patients to evaluate the awareness and acceptance of dental implants as a treatment modality for replacing missing teeth. The dental patients were selected for ease of access and to increase the response rate and can be approached during their dental visits, and most of the patients attending were from low socioeconomic status. As displayed in the result, more than half of the study population (74.8%) had heard about dental implants. This result was similar to the previous study done in Kuwait (96.4%) [14] and in Iran (76.7%) [15], while it differs significantly from the one done in Dahran Nepal (50.6%) [11]. This ranges from poor financial status and level of education in people.

More than one-third of participants (39.6%) thought that the dental implants are fixtures to the jaw bone, which was consistence with Parmita et al. (24.5%) [11] and in disagreement with AlQahtani (56.6%) [1]. Regarding the need for special care and hygiene, it was found that 27.8% of participants believed that implants needed more care than natural teeth, which was similar to a study conducted by AlQahtani (58.3%) [1], and Esfahani et al. (47.4%) [15].

In this study, 34.5 percent of patients received their initial knowledge on implants from their dentist, compared to 12.3 percent who got it online and 24.9 percent who got it from friends or family. Studies from other nations have produced contrasting findings: just 23.3 percent of Kuwaitis learned about dental implants through their dentist, while 17.7 percent of Nepalese people got their information from friends before their family dentist [11, 14]. In contrast, a poll conducted in Iran found that the dentist (40.7 percent) was the most common source of knowledge, followed by family and friends (17 percent ). Although, other reports showed that for 68% of those questioned, the main source of information about dental implants was the dentist, followed by print media (23%) and friends and acquaintances (22%) [7, 16]. It has been shown that US dentists have contributed little to bringing awareness about dental implants. Similarly, in Japan, a study showed that dentists provided no more than 20% of the information about dental implants [16].

A recent study has shown that some negative reports about dental implants have been provided in public media such as newspapers and television [5]. The idea of a permanent implant is also widely promoted by the media, which raises patients' expectations beyond what is feasible [17]. Dental education must include appropriate implantology courses to give dentists the knowledge they need to provide appropriate and realistic implant knowledge. Additionally, dentists should actively participate in educating and counseling prospective implant patients to ensure accurate scientific information.

The majority of the participants in this study (70.9%) were interested to know about dental implants, similar to a study done in India (69.9%) [10]. Though, the percentage of the population interested was less than that of a study done in Saudi Arabia (90.1%) [1]. This suggests that dental education and training about dental implants are of the utmost importance. An increasingly common and highly successful treatment option for tooth replacement is implant-supported restoration. The cost was the primary deterrent from accepting a potential implant treatment in a Swedish study, followed by surgical procedure anxiety. A qualitative method found that older patients' implant rejection was caused by their fears of pain, complications, and social shame [11]. The majority of participants in the research believed that dental implants were only for wealthy individuals and were thus pricey.

Patients' awareness of dental implants can aid in the elimination of any incorrect or negative image of the procedure that may have arisen due to a lack of adequate information. Although implants may be the best treatment option for partially or completely edentulous patients, implant therapy is not widely available in the developing world [11]. It is critical to outline the potential limitations of the current study when interpreting the findings.

## Conclusions

Bearing the result of the survey, as dentists were the main sources of information concerning dental implants, additional efforts must be made on their part and the governing bodies to take necessary measures to raise awareness amongst the population. As most patients found dental implant treatment to be expensive and unaffordable, efforts by insurance companies and respective health authorities should be made to reduce the cost of dental implants. The dentist as a professional play the main role in this regard and this can be achieved by executing patient education programs and counseling centers on dental implant usage and advantages.

## Appendices

Questionnaire Performa

Part A: Demographic analysis

Name:

Phone no./ E-mail-Id:

Age:

     under 30 years

     30-50 years

     >50 years

Gender:

     Male

     Female

Educational level:

     High school or below

     Diploma holder

     Bachelor’s degree

     Master’s degree

     PHD

Occupation:

Part B: Knowledge, awareness, and attitude regarding dental implants

1. Do you have any history of extraction?

     A. Yes

     B. No

     C. Don’t Remember

2. If yes, what was the reason for extraction/loss of tooth?

     A. Tooth decay

     B. Gum disease

     C. Accident/trauma

     D. If other, specify: 

3. When did you get the tooth extracted?

     A. 0-5 years

     B. 5-10 years

     C. 10-15 years

     D. >15 years

4. Do you want to replace the missing tooth?

     A. Yes

     B. No

5. What replacement do you need in the extracted tooth region?

     A. RPD

     B. FPD

     C. Implants

     D. Don’t know

6. Have you ever heard anything related to implants?

     A. Yes

     B. No

7. What were your first source of information about implants?

     A. Dentist

     B. Relatives & friends

     C. Family doctor/ physician

     D. Television/ radio

     E. Internet

     F. Newspaper/ magazine/ pamphlets

     G. Multimedia

8. What are the reasons for not taking dental implant as a treatment option for replacing missing teeth?

     A. Very expensive treatment

     B. Takes long time

     C. Fear/anxiety

     D. Needs surgery

     E. Lack of information about Implants

9. According to you, what do you estimate as the functional life of implants?

     A. <10 years

     B. 10-20 years

     C. 21-25 years

     D. >25 years

     E. No idea

10. Up to which amount are you prepared to pay as an additional payment for implant?

     A. 5000

     B. 10,000-20,000

     C. >20,000

     D. Varies from brand to brand (Commercial availability of implant)

11. What do you anticipate oral hygiene for the care of implants as compared with natural teeth?

     A. More than natural teeth

     B. Less than natural teeth

     C. Similar to natural teeth

     D. Can’t say

12. What are you expecting after getting treatment with implant as an option of missing teeth?

     A. Long lasting

     B. Better esthetics

     C. Less maintenance

13. Would you be interested in knowing more about dental implants?

     A. Yes

     B. No

14. Where do you think implants are anchored?

     A. Gums

     B. Teeth

     C. Jawbone

     D. Can’t say

15. Would you go ahead and restore missing teeth with dental implants?

     A. Yes

     B. No

16. Have you ever heard about experiences with implants from your friends?

     A. Yes

     B. No

17. If yes, how successful was the implant?

     A. Successful

     B. Partially successful

     C. Not successful

18. Have you ever heard about effects of dental implants on systemic health?

     A. Yes

     B. No

     C. Can’t say
